# The management of very low-calorie ketogenic diet in obesity outpatient clinic: a practical guide

**DOI:** 10.1186/s12967-019-2104-z

**Published:** 2019-10-29

**Authors:** Giovanna Muscogiuri, Luigi Barrea, Daniela Laudisio, Gabriella Pugliese, Ciro Salzano, Silvia Savastano, Annamaria Colao

**Affiliations:** Dipartimento di Medicina Clinica e Chirurgia, Unit of Endocrinology, Federico II University Medical School of Naples, Via Sergio Pansini 5, 80131 Naples, Italy

**Keywords:** Very low-calorie ketogenic diet (VLCKD), Obesity, Type 2 diabetes mellitus, Diet, Nutritionist

## Abstract

The epidemic of obesity is growing steadily across the whole world. Obesity is not only a merely aesthetic disease but is the “mother” of most chronic diseases such as associated with a range of type 2 diabetes, cardiovascular disease, obstructive sleep apnea, and cancer. However, although there is a need to find a strategy to stop this epidemic disease, most of the times the current nutritional strategies are not effective in weight loss and in long term weight maintenance. Very low-calorie ketogenic diets (VLCKD) is increasingly establishing as a successful nutritional pattern to manage obesity; this is due to rapid weight loss that gives rise to a positive psychological cycle which in turn increases the compliance to diet. Another important key point of VLCKD is the ability to preserve fatty free mass which is known to play a role of paramount importance in glucose metabolism. Despite the clinical evidence of VLCKD there are paucity of data regarding to its management. Therefore, we will provide a useful guide to be used by nutrition experts taking care of subjects with obesity. In particular, we will report recommendations on the correct use of this therapeutic approach for weight loss and management of side effects.

## Introduction

Growing evidence reported that obesity is reaching epidemic proportions. It has been reported that in 2008, over 200 million men and nearly 300 million women aged 20 and over were obese, and 65% of the world’s population live in countries where overweight [1]. Obesity could be defined as the silent killer; in fact, it significantly increases the risk of contracting diseases, such as: arterial hypertension, dyslipidemia, type 2 diabetes mellitus (T2DM), coronary heart disease, cerebral vasculopathy, gallbladder lithiasis, arthropathy, ovarian polycytosis, sleep apnea syndrome, and some neoplasms [2, 3]. In order to reach weight loss, one of the most important challenge in the management of obesity is reducing energy intake and increasing energy output. Although several strategies has been developed to reach this goal, this disorder is increasing in prevalence. The most common used nutritional pattern is characterized by an increase in complex/raw carbohydrate and a reduction in fat intake [4]. The scarce compliance of people with obesity to diet is mostly due to their preference to highly processed foods containing simple sugars rather than complex/raw carbohydrates. This is due because high glycemic index food is able to stimulate serotonin secretion that in turn provides a feeling of well being and favouring the onset of carbohydrates craving [4]. Although new anti-obesity drugs is continuously coming up, they still have some limits such as non trivial costs, potential side effects and contraindications that do not make them suitable for all people with obesity [5, 6]. In addition bariatric surgery has been demonstrated to be a useful tool for weight loss and remission of T2DM and metabolic syndrome; however, there are several complications and sequelae related to surgery and it is restricted to the obese people that do not have contraindications to surgery [7]. In this scenario very low carbohydrate ketogenic diets (VLCKDs) have been recently proposed as an attractive nutritional strategy for the obesity management in individuals who have already attempted to lose weight with diet with more equilibrated distribution of macronutrients without reaching the target weight loss. VLCKD consist of 90% of calories from fat and only 10% from carbohydrates and proteins, resulting in a highly restricted diet [8]. The benefits of VLCKDs have been demonstrated on body composition, metabolic profile, and inflammation and oxidative stress genes expression in people with obesity [9]. Merra et al. randomized people with obesity to three VLCKD protocols in which the daily kcal amount were calculated subtracting to the estimated basal metabolism 1000 kcal/day and the number of carbohydrates were < 50 g/day. However, in VLCKD1 subjects reached the half of the amount of daily protein using synthetic aminoacid supplementation containing whey protein (13.42/bag), carbohydrate (0.03/bag), fat (0.15/bag), isoleucine (0.31/bag), ornithine alpha-ketoglutarate (0.25/bag), l-citrulline (0.25/bag), taurine, (0.25/bag), l-tryptophan (0.05/bag), potassium citrate (0.45/bag), for a total of 64 kCal (268 kJ). The powder of aminoacid is dissolved in water and drunk at breakfast and lunch or dinner. In VLCKD2 and 3 the composition of macronutrient was the same of VLCKD1 while there was a different source of carbohydrate i.e. < 35 g; > 80% from simple sugars and < 30 g; > 35% from complex sugars, respectively. VLCKDs protocol resulted in weight loss and an improvement of metabolic profile. In addition, after VLCKD with synthetic aminoacidic protein replacement (VLCKD1) there was a significant modulation of superoxide dismutase (SOD)-1 gene expression along with a reduction or C-reactive protein, thus suggesting the efficacy of VLCKD with synthetic aminoacidic protein replacement, for the reduction of cardiovascular risk, without the development of sarcopenia and activation of inflammatory and oxidative processes [9]. Regarding gene expression Garbow et al. reported that, in C57BL/6J mice, VLCKD determines a reduction, up to the suppression, of the expression of inflammatory cytokines and chemokines, as well as the production of reactive species oxy-hydrogen (ROS) [10]. Mutations in the gene encoding the enzyme copper/zinc (Cu/Zn) SOD1 were the first mutation identified to be associated with familial amyotrophic lateral sclerosis (ALS). Further, it has been demonstrated that VLCKD in the G93A-SOD1 transgenic mice model of familial amyotrophic lateral sclerosis promotes ATP synthesis and neuroprotection [11]. Ketogenic diets induce a metabolic condition called “physiological ketosis” by Hans Krebs which is different from the pathological diabetic ketosis [12]. In the past the ketogenic diet has been used as treatment of various diseases such as pediatric pharmacoresistant epilepsy [13]. Recently, VLCKDs have undoubtedly demonstrated to be an effective tool to tackle obesity [14], dyslipidemia and most of obesity-related cardiovascular risk factors [15, 16]. The rapid initial weight loss is due to natriuresis and diuresis resulting from the decrease in insulin levels and increase in glucagon levels and ketone production [17, 18]. Even after the initial diuresis, the rate of weight loss remains faster than with other types of diet because the calorie level is so low. Further, because the nutritional pattern is unfamiliar and the diet is perceived to be temporary, patients may have a higher compliance rate than on nutritional patterns that require a longer time to lose the same amount of weight. The relative preservation of protein mass also is an advantage, certainly as compared with starvation [19]. Given the growing use of VLCKDs in the management of obesity, we will provide a practical guide on its clinical indications and contraindications and on the steps involved in ketogenic diet initiation, monitoring, and management of its side effects in outpatient clinic.

## Very low-calorie ketogenic diet protocol

VLCKD is a nutritional protocol that resembles fasting through a marked restriction of daily carbohydrate intake, usually lower than 30 g/day (≃ 13% of total energy intake) along with a relative increase in the proportions of fat (≃ 44%) and protein (≃ 43%) and a total daily energy intake < 800 kcal [20]. The VLCKD protocol is a weight loss nutritional program based on a high-biological-value protein (coming from milk, peas, whey and soy) preparations diet and natural foods. Each protein preparation contains 18 g protein, 4 g carbohydrate, 3 g fat (mainly high-oleic vegetable oils) and provides approximately 100–150 kcal. This protocol is divided in three stages: active, re-education, and maintenance.

### Active stage

The active stage is characterized by a very low-calorie diet (600–800 kcal/day), low in carbohydrates (< 50 g daily from vegetables) and lipids (only 10 g of olive oil per day). The amount of high-biological-value proteins ranged between 0.8 and 1.2 g per each Kg of ideal body weight in order to preserve lean mass and to meet the minimal daily body requirements. This stage is further divided in 3 ketogenic phases: in phase 1, the patients eat high-biological-value protein preparations five times a day, along with vegetables with low glycemic index. In phase 2, one of the protein servings is replaced by natural proteins such as meat/egg/fish either at lunch or at dinner. In the phase 3, a second serve of the natural protein low in fat replaced the second serve of biological protein preparation. Being a very low caloric nutritional pattern, it is recommended to supplement patients with micronutrients (vitamins, such as complex B vitamins, vitamin C and E, minerals, including potassium, sodium, magnesium, calcium; and omega-3 fatty acids) according to international recommendations. This active stage is kept until the patient loses most of weight loss target, about 80%. Therefore, the ketogenic phases are variable in time depending on the individual and the weight loss target. The active stage generally lasts between 8 and 12 weeks in total.

### Re-education stage

After the ketogenic phases, the patient is switched to low-calorie diet. At this point, the patients will progressively reintroduce different food groups and in the meantime participates in a program of alimentary re-education in order to maintain weight long term. Carbohydrates are gradually reintroduced, starting from foods with the lowest glycemic index (fruit, dairy products—Phase 4), followed by foods with moderate (legumes—Phase 5) and high glycemic index (bread, pasta and cereals—Phase 6). The daily calorie intake in the reintroduction period (Phases 4–6) ranges between 800 and 1500 kcal/day. After the reintroduction of food there is a maintenance stage which includes an eating plan balanced in carbohydrates, protein, and fat. The main target of this stage is to keep lost weight and to promote healthy lifestyle. In this stage the calories consumed ranged between 1.500 and 2.000 kcal/day, depending on individual.

## Indications and contraindications

The The European Association for the Study of Obesity (EASO) guidelines defines as very low calorie diets (VLCD) a diet that usually provide less than 800 kcal/day and highlights as it may be used only as part of a comprehensive programme under the supervision of an obesity specialist or another physician trained in nutrition and dietetics. The prescription of VLCD should be limited for specific patients and for short frametime. VLCDs are unsuitable as a unique source of nutrition for children and adolescents, pregnant or lactating women and the elderly [21]. According to the National Institute for Health and Care Excellence (NICE) guidance, VLCD should be considered as part of a multistrategical weight management for people who are obese and who have a clinically assessed need to lose weight rapidly (for example, those who need joint replacement surgery or who are seeking fertility services). VLCD should be is followed for a maximum of 12 weeks (continuously or intermittently) with ongoing clinical Support [22]. The VLCKDs indications of ADI (Associazione Italiana di Dietetica e Nutrizione Clinica) are the following [23]:

1. Morbid obesity or complicated (T2DM, dyslipidemia, hypertension, metabolic syndrome, obstructive sleep apnoea syndrome (OSAS), bone diseases or severe arthropathy);
2. Severe obesity with bariatric surgery indication (in the preoperative period);
3. Patients with severe comorbidities needing a rapid weight loss;
4. Non-alcoholic fatty liver disease (NAFLD);
5. Drug-resistant epilepsy.

The VLCKDs controindications of *Associazione Italiana di dietetica e Nutrizione Clinica* (ADI) are represented by:

1. Pregnancy and lactation;
2. History of mental disorders and behavioral problems, abuse of alcohol and other substances;
3. Hepatic or renal failure;
4. Type 1 Diabetes;
5. Porphyria, unstable angina, recent myocardial infarction (Table 1).

**Table 1:** Indications and contraindications to VLCKD of ADI (Associazione Italiana di Dietetica e Nutrizione Clinica) and SIE (Società Italiana di Endocrinologia)

	ADI	SIE
Indications	Morbid obesity or complicated (type 2 diabetes, dyslipidemia, hypertension, metabolic syndrome, OSAS, bone diseases or severe arthropathy) Severe obesity with bariatric surgery indication (in the preoperative period) Patients with severe comorbidities needing a rapid weight loss Non-alcoholic fatty liver disease (NAFLD) Drug-resistant epilepsy	Severe obesity Management of severe obesity before bariatric surgery Sarcopenic obesity Obesity associated with type 2 diabetes (preserved beta cell function) Obesity associated with hypertriglyceridemia Obesity associated with hypertension Pediatric obesity associated with epilepsy and/or with a high level of insulin resistance and/or comorbidities, not responsive to standardized diet
Contraindications	Pregnancy and lactation History of mental disorders and behavioral problems, abuse of alcohol and other substances Hepatic or renal failure Type 1 Diabetes Porphyria, unstable angina, recent myocardial infarction	Type 1 diabetes mellitus Latent autoimmune diabetes in adults β-cell failure in type 2 diabetes mellitus Use of sodium/glucose cotransporter 2 (SGLT2) inhibitors (risk for euglycemic diabetic ketoacidosis) Pregnancy and breastfeeding Kidney failure and moderate-to-severe chronic kidney disease Liver failure Hearth failure (NYHA III-IV) Respiratory failure Unstable angina, recent stroke or myocardial infarction (or myocardial infarction (or myocardial infarction (< 12 months) Cardiac arrhythmias Eating disorders and other severe mental illnesses, alcohol and substance abuse Active/severe infections Frail elderly patients 48 h prior to elective surgery or invasive procedures and perioperative period Rare disorders: porphyria, carnitine deficiency, carnitine palmitoyltransferase deficiency, carnitine-acylcarnitine translocase deficiency, mitochondrial fatty acid β-oxidation disorders, pyruvate carboxylase deficiency

In 2016, VLCKD has also been reported with similar indications in the standards of care in obesity released by the Italian Society of Obesity (SIO) and ADI itself [24]. The recent consensus statement from the Italian Society of Endocrinology (SIE) strongly recommended VLCKDs in:

1. Severe obesity;
2. Management of severe obesity before bariatric surgery;
3. Sarcopenic obesity;
4. Obesity associated with T2DM (preserved beta cell function);
5. Obesity associated with hypertriglyceridemia;
6. Obesity associated with hypertension;
7. Pediatric obesity associated with epilepsy and/or with a high level of insulin resistance and/or comorbidities, not responsive to standardized diet.

There is a weak recommendation for:

1. Obesity associated with dysbiosis of the gut microbiota;
2. Obesity associated with high levels of LDL-cholesterol and/or low levels of HDL-cholesterol;
3. Obesity associated with non-alcoholic fatty liver disease (NAFLD);
4. Obesity associated with heart failure (NYHA I–II);
5. Obesity associated with atherosclerosis;
6. Male obesity secondary hypogonadism;
7. Obesity associated with polycystic ovary syndrome (PCOS);
8. Menopausal transition-related obesity;
9. Neurodegenerative disorders associated with sarcopenic obesity.

The absolute contraindications are represented by type 1 diabetes mellitus, latent autoimmune diabetes in adults, β-cell failure in T2DM, use of sodium/glucose cotransporter 2 (SGLT2) inhibitors (risk for euglycemic diabetic ketoacidosis), pregnancy and breastfeeding kidney failure and moderate-to-severe chronic kidney disease, liver failure, hearth failure (NYHA III–IV), respiratory failure unstable angina, recent stroke or myocardial infarction (< 12 months), cardiac arrhythmias, eating disorders and other severe mental illnesses, alcohol and substance abuse, active/severe infections, frail elderly patients, 48 h prior to elective surgery or invasive procedures and perioperative period, rare disorders: porphyria, carnitine deficiency, carnitine palmitoyltransferase deficiency, carnitine-acylcarnitine translocase deficiency, mitochondrial fatty acid β-oxidation disorders, pyruvate carboxylase deficiency (Table 1) [20]. Finally according to *Società Italiana di Chirurgia dell’OBesità e delle malattie metaboliche* (SICOB) the use of VLCKD from 15 to 30 days prior to surgery allows to get satisfactory results in less time, with less money and fewer side effects than the intragastric balloon [25].

## Efficacy and management of the most common side effects

### Efficacy

The VLCKD is a nutritional protocol that provides suddenly beneficial effects on anthropometric and metabolic parameters and on body composition [9]. The assessment of anthropometric measurements (BMI, weight, waist circumference and hip circumference), body composition and hydration status (by bioelectrical impedance analysis) is recommended at baseline, during the active state and at the end of the VLCKD program. In order to investigate the efficacy of VLCKD on metabolic parameters glucose, insulin, total cholesterol, HDL-cholesterol, LDL-cholesterol, triglycerides (serum) should be assessed at baseline and at the end of the VLCKD program (Table 2).

**Table 2:** Anthropometric measurements and laboratory assessment to be monitored during the VLCKD

	Parameters	Baseline	During active stages	At the end of VLCKD
Anthropometric assessment	Weight, height, BMI	✓	✓	✓
	Body composition and hydration status (by bioelectrical impedance analysis)	✓	✓	✓
Laboratory assessment	Complete blood count with platelets	✓	✓	✓
	Sodium, potassium, magnesium, and inorganic phosphate	✓	✓	✓
	Serum liver and kidney tests (including albumin, AST, ALT, blood urea nitrogen, creatinine, γ-GT, total and direct bilirubin)	✓	✓	✓
	Fasting lipid profile	✓		✓
	25(OH)D, calcium	✓		✓
	Glucose, Insulin	✓		✓
	β-Hydroxybutyrate (capillary blood or urine)		✓	
	TSH, FT4	✓		
	Complete urinalysis and microalbuminuria (urine)	✓	✓	✓

*BMI* body mass index, *AST* aspartate aminotransferase, *ALT* alanine transaminase, *γGT* γ-glutamyltransferase, *25(OH)D* 25-hydroxy vitamin D, *TSH* thyroid-stimulating hormone, *FT4* free thyroxine

#### Short term side effects

##### Dehydration

Dehydration is the most common early-onset complication of VLCKD. Signs and symptoms of dehydration are mostly represented by dry mouth, headache, dizziness/orthostatic hypotension and visual disturbance [26]. Therefore, proper water intake (at least 2 L of sugarless fluids daily) is mandatory mostly in the first 3 phases. In order to relieve headache, it is advisable to take mild analgesics as pills instead of liquid formulations because they could contain sugar. However, it should notice that headache is a short term, temporary side effect; in fact, VLCKDs are currently used in the treatment of chronic migraine [27]. Electrolyte abnormalities such as hyponatremia and hypomagnesemia, which are potentially due to dehydration, urinary excretion of ketone bodies and poor intake of micronutrients, could occur mostly in the active stage. It has been reported that in the sodium-equilibrated subjects on a constant sodium intake, the natriuresis of early starvation is transient and lasts typically from days 2 through 6 of the fast, the peak natriuresis occurs with some individual variation on day 4 of the fast. Following the natriuresis, there is a return to positive sodium balance, which will be kept for the duration of fasting. In contrast to the natriuresis, the small and variable kaliuresis that accompanies starvation occurs on days 5 through 7 of the fast, after which there is a return to positive potassium balance [17]. If patient complain hypotension-related symptoms, it is advisable to increase salt intake wherever there are no contraindications. Supplementing with magnesium can help reduce muscle cramps, difficulty sleeping and irritability mostly in the active stage.

##### Hypoglycemia

Transient hypoglycemia could be a complication of the VLCKD, usually in the initial period of protocol [28]. The majority of the glucose lowering effect has been related to calorie restriction, whereas weight loss has an increasing contribution over the time through the decrease in intraabdominal (visceral) adipose tissue. Further, It has been demonstrated that ketone bodies can stimulate insulin secretion in normal humans [29]. The reduction of fat mass consequent to weight reduction during VLCKD is associated with decreased oxidation of lipids and increased oxidation of glucose. The net effect of the shift in oxidation of fuels was enhanced glucose metabolism and improved insulin sensitivity [30].

The reduction in carbohydrate intake is associated with an early and significant decrease in hepatic triacylglycerol content that in turn suppresses hepatic glucose production improving hepatic insulin sensitivity [31]. However, most patients experiencing transient hypoglycemia recover without assistance and do not show hypoglycemic symptoms. If blood glucose is less than 40 mg/dL and hypoglycemia is symptomatic, it is suggested the assumption of carbo-hydrate-containing beverages such as orange juice.

##### Lethargy

Transitory lethargy could occur in the first days of the protocol and it occurs as the body switches from burning carbohydrates to burning fat for energy. However, if lethargy persists more than few days, medical investigations are recommended, as lethargy could be also a symptom of dehydration, excessive ketosis and nutrient deficiencies. It is also recommended to measure ketonemia/ketonuria and eventually, it is suggested the assumption of carbo-hydrate-containing beverages such as orange juice.

##### Halitosis

Halitosis can occur whilst VLCKD. This is due to ketosis and generally it is caused by an increase in acetone levels. This is characteristic of VLCKD and it could be considered as an additional sign of being in ketosis. The halitosis will only last whilst they are following the active stage; chewing on a low-calorie mint or sugar free chewing gum is recommended to manage it.

##### Gastrointestinal side effects

The most common early complications of VLCKD are represented by gastrointestinal disturbances, involving nausea/vomiting, diarrhea, or constipation. Gastrointestinal (GI) disturbances are often related to scarce tolerance of the diet that result in a significant resistance to the ketogenic diet and even blunting its efficacy. Diarrhea is the most common of these symptoms, but most cases is transient and easily controlled, sometimes using short-term antidiarrhea medication. This is could due to defective absorption and intolerance of fat. In addition, the high-lipid diet ketogenic diet’s high-fat content prolongs the gastric emptying time thus favouring gastroesophageal reflux disease, nausea and vomit. A modification of the diet menu such as frequent intake of small amounts, intermittent use of GI drugs such as antiemetics, GI tract regulators, and antacids. Constipation might be caused by a decreased intake of fiber and/or by a decreased volume of food [32]. Constipation can be successfully controlled ensuring an adequate fluid intake and/or using low-calorie bulk laxative and/or intermittent enemas. The supplement of dietary fibre may improve constipation increasing the number of bowel movements. In subjects with pre-existing constipation, diverticular disease or haemorrhoids an extra dietary fibre (psyllium 3.5 g twice daily is recommended) from the beginning of the diet need to be considered [33]. Acute pancreatitis is a rare but serious complication that is often fatal [34]. Pancreatitis can be caused by hypertriglyceridemia [35]. Hepatitis is also a rare complication that could be fatal [28]. Both these conditions may occur more often if there is the concomitant use of antiepileptic drugs [36]. Discontinuation of the VLCKD and adequate supportive treatment are required for successful recovery.

##### Hyperuricemia

Serum uric acid is known to increase in individuals on ketogenic regimens providing less than 900 calories per day. Plasma uric acid levels increase on VLCKDs, especially if the diet is very low in carbohydrate. Uric acid also follows a biphasic course having a peak in 1 to 2 weeks and then decreases toward baseline [19]. Patients with a prior history of gout may be more prone to develop exacerbations. However, attacks of acute gouty arthritis, has been described in less than 1% of subjects following VLCKD [37], (Table 2).

#### Long term side effects

##### Hypoproteinemia

Hypoproteinemia could occur probably as a consequence of gluconeogenic consumption due to carbohydrate restriction [38]. In order to manage this side effect, it is recommended to increase protein intake from 1 g/kg/day to 1.5 g/kg/day while the lipid-to-nonlipid ratio is kept.

##### Hypocalcemia and bone damage

It has been reported that serum ionized calcium, as well as total serum calcium, plasma parathyroid hormone (PTH) and calcitonin levels remain stable even during the 4-week long VLCD [39]. In particular calcium balance has been reported to be positive in people with obesity undergoing a moderate VLCKD taking high calcium intake (1200 mg/day); the retention of ingested calcium was proportional to the amount of carbohydrate in the diet [40]. Although calcium metabolism seems to be preserved in VLCKD, few evidence reported that very low calorie diet has a negative effect on both bone mineral content (BMC) and bone mineral density (BMD), in particular in the femoral neck and greater trochanter, and that this effect is proportional to the degree of reduction in body weight, as well as in fat and lean mass [41, 42]. However, there are no data to suggest this increases long-term fracture risk. Although no studies have been carried out in VLCKD, diet high in acid-ash proteins have been described to be associated to excessive calcium loss because of its acidogenic content. Calcium is provided as buffer from the skeleton through the active resorption of bone; indeed, calciuria is directly related to net acid excretion and it is not compensate by increasing intestinal calcium absorption [43]. Thus, taken together, all these observations raise some concern about the risk of a moderate loss of bone mineral content during VLCD. To prevent such a consequence of dieting, it is recommended to provide an adequate high intake of calcium and vitamin D, as well as an appropriate amount of carbohydrate.

##### Lipid profile changes

The effects of VLCKD on plasma lipoproteins in obese patients is characterized by a fall in plasma triglycerides, an increase in LDL-cholesterol and a neutral effect on HDL-cholesterol. The prolonged ingestion of high lipid diets could be responsible of increase in LDL cholesterol [20]. However, this seems to be a transient effect as demonstreated by the reabsorption of ateroma produce by ketogenic diet, after returning to a normal diet [44]. Since the increase in LDL has been reported to spontaneously improve, the decrease of the lipid-to-nonlipid ratio to 3:1 or the use of cholesterol-reducing medication should be taken into account if LDL does not normalize after returning to normal diet.

##### Urolithiasis

Urolithiasis is another possible complication of the VLCKD [45, 46]. The stones are mostly are mostly made of uric acid, calcium oxalate, or a mixture of calcium oxalate and calcium phosphate/uric acid. [45, 46]. The cause of VLCKD—related urolithiasis are represented by chronic acidosis, dehydration, and fat malabsorption. Risk factors of developing urolithiasis include young age, family history of kidney stones, and a urine Ca/Cr ratio of > 0.2 [45]. In order to prevent the onset of urolithiasis it is suggested to recommend an adequate daily fluid intake (at leat 2 L) and to alkalinize urine using oral potassium citrate.

##### Gallstones

The low fat content and/ore the rapid weight loss increases the risk of developing gallstones. In fact it has been already reported that rapid weight loss, either by VLCD or bariatric surgery, is a known risk factor for gallstone formation [47]. This is due to the supersaturation of bile with cholesterol, leading to cholesterol crystallization and stone formation, and to the insufficient gallbladder emptying caused by blunted due to impaired motility. Both mechanisms happen in VLCD: supersaturation is mostly due to decreased bile salt levels and increased cholesterol levels whilst impaired motility is due to reduced gallbladder stimulation because of the low-fat content [48, 49]. In order to prevent the risk of gallstones, a fat intake of 7–10 g per day has been reported as a threshold for maintaining an efficient gallbladder emptying [50].

##### Hair loss

Hair loss occurs mostly in patients in whom weight loss is associated with the loss of body cell mass (e.g., a significant negative nitrogen balance). When mobilized body protein plus dietary protein are not enough to meet requirements, the low priority of hair growth for available protein accounts for the telogen effluvium [51]. The hair loss is transient and hair grows back well as weight stabilizes. However, an increase in protein intake during fasting in order to preserve nitrogen balance, contribute to eliminate almost completely hair loss, (Table 2).

## Conclusions

VLCKD is an ideal therapeutic tool for people with obesity and in particular for that subjects who have already experienced unsuccessful diet in the past and/or have urgently need to lose weight (people with obesity with joint diseases, people with obesity with bariatric surgery indications, people with obesity with cardiovascular risk factor etc.). Given the potential of VLCKD in determining remission of T2DM, VLCKD should be also taken into account in people with obesity with short T2DM duration.

**Fig. 1:**
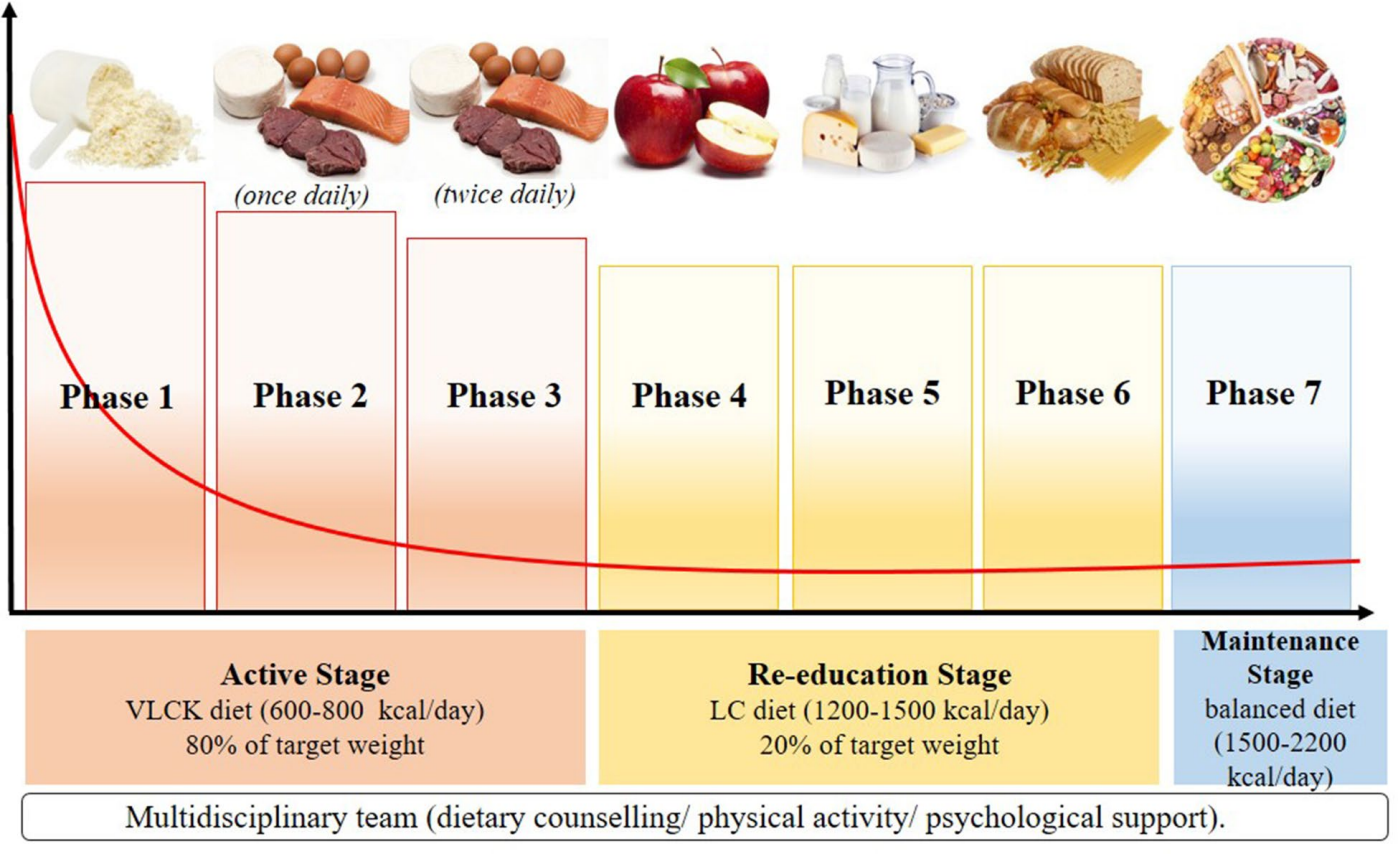
Scheme of the stages of VLCKD

Once weight goal is achieved, it is mandatory to suggest an appropriate healthy lifestyle (physical activity and a balanced nutritional pattern such as Mediterranean Diet) for long-term body weight maintenance. The scheme of the stages of VLCKD is reported in Fig. 1.

## Data Availability

Not applicable.

## Abbreviations

T2DM: type 2 diabetes mellitus
VLCKDs: very low-calorie ketogenic diets
ADI: Associazione Italiana di Dietetica e Nutrizione Clinica
NAFLD: non-alcoholic fatty liver disease
SIO: Italian Society of Obesity
SIE: Italian Society of Endocrinology
LDL: low-density lipoprotein
HDL: high-density lipoprotein
PCOS: polycystic ovary syndrome
SICOB: Italian Society of Bariatric Surgery and Metabolic Diseases
PTH: parathyroid hormone
BMC: bone mineral content
BMD: bone mineral density
